# Evaluation of *Echinochloa frumentacea* under saline–alkaline conditions and its comparison with five forage species

**DOI:** 10.1093/aobpla/plaf066

**Published:** 2025-11-20

**Authors:** Xueqin Wang, Qingxia Zhang, Fengxia Li, Qiaoli Ma, Bo Zhang, Fengju Zhang

**Affiliations:** Ningxia Institute of science and technology development strategy and information, Ningxia Hui Autonomous Region, 750021, China; School of Forestry and Grassland Science, Ningxia University, Yinchuan, 750021, China; Institute of Agricultural Resources and Environment, Ningxia Academy of Agricultural and Forestry Sciences, Yinchuan, 750021, China; School of Forestry and Grassland Science, Ningxia University, Yinchuan, 750021, China; National Wolfberry Engineering Research Center, Ningxia Academy of Agricultural and Forestry Sciences, Yinchuan, 750021, China; School of Ecology and Environment, Ningxia University, Yinchuan, 750021, China

**Keywords:** *Echinochloa frumentacea*, saline–alkaline soil, root morphology, antioxidant enzymes, forage crops, salinity tolerance

## Abstract

Salinity is one of the most devastating abiotic stresses limiting crop productivity. Here, the salinity tolerance level and physiological changes in *Echinochloa frumentacea* in saline and alkaline soils were estimated by studying root morphology, quantifying ions (Ca^2+^, K^+^, Na^+^, Ca^2+^/Na^+^, and K^+^/Na^+^) in roots, and measuring antioxidant enzyme activities, malondialdehyde (MDA), proline, and soluble sugar contents. *Echinochloa frumentacea* was tested against four neutral and alkaline salts, NaCl: Na_2_SO_4_:NaHCO_3_:Na_2_CO_3_ in different proportions at 60, 120, 180, 240, and 300 mmol L^−1^ concentrations. *Echinochloa frumentacea* was evaluated and compared with plant species, which are commonly cultivated in non-saline and alkaline soils i.e. *Echinochola crusgalli*, *Avena sativa*, *Salicornia europaea*, *Medicago sativa*, and *Glycyrrhiza uralensis*. The results revealed an increase in root length, diameter, absorption area, fresh, and dry weight at 120 mmol L^−1^. However, a gradual decrease in these parameters was observed at higher salt concentrations. In contrast, an increase in superoxide dismutase (SOD), peroxidase (POD), catalase (CAT) activities, and MDA and proline levels were observed with increasing salt concentration. The roots of *E. frumentacea* absorbed higher levels of ions than the other five forage plant species. Higher K^+^/Na^+^ and strong root structure in *E. frumentacea* indicate its better tolerance in saline soil than in alkaline soil. Our results demonstrate that *E. frumentacea* can tolerate up to 120 mmol L^−1^ salt in a saline–alkaline environment and is more suitable for growth in saline soil. In addition, the root system of *E. frumentacea* can be used to dechlorinate the chloride from soil and reduce its toxic effect on plants. It can also be used as a target species for selection and breeding programs to improve salt tolerance in future studies.

## Teaser

Our research identifies Echinochloa frumentacea as a salt-tolerant forage crop. In saline-alkaline conditions, E. frumentacea can survive under moderate salt levels (up to 120 mmol ^−1^), by developing a stronger and more extensive root system. Its roots effectively absorb and manage sodium ions while maintaining a healthy K^+^/Na^+^ ratio. Moreover, it activates antioxidant defenses to tolerate salt-induced stress. We conclude that E. frumentacea is a well-suited plant for cultivating and rehabilitating saline soils. It serves as a good candidate for breeding programs aimed at enhancing salt tolerance in other crops and can help detoxify chloride-affected land.

## Introduction

Salinity is one of the most devastating environmental factors limiting the plant's normal growth and productivity because most crop plants are sensitive to high salt concentrations in the soil (Hasanuzzaman et al. 2013). NaCl, Na_2_SO_4_, NaHCO_3_, and Na_2_CO_3_ are the major harmful salts derived from neutral salts and alkaline salts in the soil. A significant amount of land in the world is being affected by salinity. More than 45 million hectares (M ha) of irrigated land which accounting for 20% of the total land area have been damaged by salt worldwide and 1.5 M ha are taken out of production every year due to high soil salinity levels in the soil (Munns and Tester 2008). China ranks third among the top 10 countries in terms of saline–alkaline land area (Li et al. 2018). The saline–alkaline land is mainly distributed in 17 provinces and autonomous regions including northwest, northeast, north China, and coastal areas. The total area of saline–alkaline wasteland and saline–alkaline land affecting cultivated land is more than 500 million mu (1 mu = 666.7 m^2^) (Liu et al. 2016). Saline–alkaline soil is also one of the main ecological and environmental problems and the main factor limiting the agricultural production and development in the Ningxia Yellow River Diversion Irrigation District. The saline–alkaline land in the Ningxia Yellow River Diversion Irrigation District accounts for a large proportion of the cultivated land in the irrigated area, among which the Yinbei area is the most serious (Wu et al. 2021). Earlier research on the utility of such saline–alkaline lands have indicated the successful cultivation of several plant species including sorghum, *Suadeda salsa*, *Salicornia bigelovii*, *Mesembryanthemum crytalinum*, wild soybean, *E. frumentacea*, *Miscanthus sacchariflorus*, and *Miscanthus lutarioriparius*, and others (Guo et al. 2020, Cheng et al. 2023, Tang et al. 2024). This has proven to be a sustainable strategy, as by cultivating salt–tolerant crop species on saline–alkaline lands, such a large reserve of land resources can be utilized to ensure food security (Yang et al. 2010).

At the molecular level, high soil salinity can affect plant metabolism by disrupting cellular homeostasis and uncoupling key physiological and biochemical processes (Tanou et al. 2009). Stress induces an osmotic imbalance and oxidative stress in plants resulting in a significant increase in reactive oxygen species (ROS), including singlet oxygen (^1^O_2_), superoxide (O_2_^−^), hydroxyl radical (^•^OH), and hydrogen peroxide (H_2_O_2_) (Ahmad et al. 2011). Plants minimize the excessive ROS activity by using both enzymatic and nonenzymatic antioxidants. In the scavenging process of ROS, superoxide dismutase (SOD), catalase (CAT), peroxidase (POD), etc are key antioxidant enzymes (Farieri et al. 2016). Other than these enzymes, malondialdehyde (MDA), a natural product of lipid peroxidation, is also an indicator of the degree of salt stress (Morales and Munné-Bosch 2019). In terms of osmolytes, proline increases in mild or acute stress conditions and acts as a nitrogen storage reservoir, which contributes to a decrease in cytoplasmic osmotic potential in inducing stress tolerance in plants (Singh et al. 2021). The effect of salt stress on plants depends on the concentration and duration of salt exposure, plant genotypes, and environmental factors (Hasanuzzaman et al. 2013). Most crops do not grow well on saline soils. The highly tolerant crops can withstand up to 10 g L^–1^ salt concentration. The moderately tolerant crops can tolerate up to 5 g L^–1^ salt concentrations. Whereas, the sensitive group can tolerate salt concentration up to 2.5 g L^–1^ (Brouwer and Heibloem 1985, Appleton et al. 1999). The impact of salt stress generally contains osmotic and ionic effects. However, alkaline stress added to the influence of high pH, can inhibit ion uptake and disrupt the ionic balance of plant cells (Kekere and Bamidele 2014, García-Caparrós and Lao 2018). When plants are cultivated in saline–alkaline soils, roots are the first organs that perceive the stress and then transmit the signals and effects to the aboveground organs (Fang et al. 2021). Therefore, the root distribution pattern in the soil is a reflection of the plant's ecological adaptation and may increase the chance of plant survival under stress (García-Caparrós and Lao 2018).

Among the major crop plants grown in China, particularly in the Hetao Ningxia Plain, *E. frumentacea* has been widely planted. Research has shown that it exhibits fast growth in saline soils. The utility of *E. frumentacea* in forage and green manure makes it a valuable resource (Chandrasekar et al. 2005). With its strong leaching ability of Na^+^ and SO_4_^2−^, it can reduce pH and total salt levels, while increasing the availability of major nutrients, ultimately leading to better plant growth (Cheng et al. 2023). However, how *E. frumentacea* adapts to such harsh saline–alkaline environments is not yet explored in detail. Moreover, since the salinity level in saline–alkaline soils is not uniform, it is essential to understand how it responds to different salinity levels. Current knowledge indicates that *E. frumentacea* develops various physiological and biochemical mechanisms to survive in soils with high salt concentrations. Principal mechanisms include ion homeostasis and compartmentalization, ion transport and uptake, biosynthesis of osmoprotectants and compatible solutes, activation of antioxidant enzyme and synthesis of antioxidant compounds, synthesis of polyamines, generation of nitric oxide, and hormone modulation (Gupta and Huang 2014). Moreover, there are limited data on how *E. frumentacea* roots respond to soil salinity. Moreover, how plant growth is impacted by salt stress is least understood. Considering these scenarios, this study aims to improve our understanding of the salt tolerance level related to seedling establishment, and the physiological and biochemical adaptation mechanisms under saline and alkaline environments in *E. frumentacea*. Another important but least explored factor is to establish the tolerance level by comparison with the crop plants, which are commonly cultivated in the non-affected soil. To this regard, we compared the salt tolerance levels of *E. frumentacea* (barnyard millet) salt tolerance levels with *Echinochola crusgalli* (barnyard grass), *Avena sativa* (oat), *Salicornia europaea* (glasswort), *Medicago sativa* (alfalfa), and *Glycyrrhiza uralensis* (Chinese liquorice). By analysing these, we discuss the overall response mechanism of *E. frumentacea* to salinity stress.

## Material and methods

### Experiment 1: Salt stress treatment of *Echinochloa frumentacea*

#### Plant material and salt stress treatments

The types of saline–alkaline land in Yinbei, Ningxia are complex. Therefore, in this study, four single salts, NaCl, Na_2_SO_4_, NaHCO_3_, and Na_2_CO_3_, were mixed in the order of increasing alkaline salt proportions. The ratios of divalent anions (CO_3_^2−^, SO_4_^2−^) to monovalent anions (HCO_3_^−^, Cl^−^) were both 1:1, and 5 mixed salt stress groups were established (A1–A5). At the same time, 5 concentration gradients (B1–B5) were set for each mixed salt group, which were 60, 120, 180, 240, and 300 mmol L^−1^, and a total of 25 mixed salt stress solutions with different salt concentrations and different pH values were simulated (Table 1). Whereas, B0 was used as a control. The salt concentrations i.e. B1–B5, were established based on previous reports on evaluation of salt stress tolerance of different plant species (Mostofa et al. 2015, Elhindi et al. 2017, Hniličková et al. 2019, Xu et al. 2022). The stress solutions were prepared in Hoagland’s nutrient solution. In addition, the same concentration of Hoagland’s nutrient solution was used as blank control. The pH value of each solution was measured using a pH meter (PHB-4, Shanghai LICHENKEYI, China).

**Table 1.** The molar ratio of salt composition under mixed salt and pH values of each treatment.

Groups	NaCl:Na_2_SO_4_:NaHCO_3_:Na_2_CO_3_	pH				
		B1 (60 mmol L^−1^)	B2 (120 mmol L^−1^)	B3 (180 mmol L^−1^)	B4 (240 mmol L^−1^)	B5 (300 mmol L^−1^)
A1	0.5:0.5:0:0	7.02	7.03	7.09	7.10	7.12
A2	0.25:0.5:0.25:0	8.27	8.4	8.46	8.45	8.44
A3	0.05:0.45:0.45:0.05	8.63	8.66	8.74	8.71	8.85
A4	0.1:0.4:0.4:0.1	9.02	9.27	9.30	9.35	9.44
A5	0.25:0.25:0.25:0.25	9.62	9.68	9.70	9.73	9.76

A1––A5 represents ratios of mixtures of salt stress groups. B1––B5 represents the concentration of each salt group.

This experiment was carried out in the artificial climate chamber of the Ecology Center of Ningxia University from April to June 2020. The temperature of the chamber was 25 ± 1 °C during the day and 20 ± 1 °C during the night, with a light cycle of 14 h/10 h. *Echinochloa frumentacea* was planted in plastic pots as three replicates and under five different salt stress treatments. At the end of the experiment, *E. frumentacea* seedlings were harvested and analyzed for root characterization to determine salt–tolerant abilities.

#### Experimental conditions and treatments

The experiment was carried out under potting method. The potting device consists of a rectangular plastic pot and a seedling tray of 530 mm × 280 mm in size. Seeds with full grains and uniform size were selected and sterilized in 4% NaClO for 15 min and then rinsed with distilled water 4–5 times. The sterilized seeds were soaked in distilled water for 24 h and then dried by placing them on filter paper. Treated seeds were sown in a seedling tray filled with vermiculite. Five seeds per hole were sown and covered with ∼0.5 cm of vermiculite. All treatments were replicated in triplicate. The seeded pots were placed in an artificial climate chamber at 25 ± 1 °C/20 ± 1 °C (day/night), and the light cycle was set to 14 h/10 h (day/night). The light intensity in the climate chamber during the experiment was 180 μmol m^−2^ s^−1^. The relative humidity levels were 35%–40%. The carbon dioxide concentration was set consistent with the outside atmosphere. After germination, only one plant per hole was left, and the seedlings were irrigated with water and Hoagland’s and nutrient solution. When the seedlings grew to 2 leaves and 1 heart, they were transplanted into plastic pots for salt stress treatment. To avoid the salt shock effect, the A1 stress solution was irrigated with a daily increase of 0.3% salt concentration, and A2, A3, A4, and A5 solutions were irrigated with a daily increase of 0.2% salt concentration, and the control was only watered with 1/2 Hoagland’s nutrient solution. After reaching the final concentration, the daily application of salt stress solution was replaced by every 2 days (during this period, the evaporated water was supplemented by the weighing method), and samples were taken after 15 days of culture for the determination of various morphological indicators.

### Experiment 2: Absorption and leaching

#### Experimental design

A two-factor random block arrangement design was adopted, and the salinity type was set as the first factor. There are two levels, namely: saline soil and alkaline soil. Pasture type was set as the second factor, with six levels, namely: *E. frumentacea*, barnyard grass, oats, glasswort, alfalfa, and liquorice (Table 2). Each treatment was repeated three times for a total of 36 treatments.

**Table 2.** Names and descriptions of plant species used in the experiment.

Specie name	Common name/family	Characteristics	References
*Echinochloa frumentacea* (Roxb.)	Barnyard millet/Poaceae	Annual herb, wide adaptability, salinity tolerance	(Cheng et al. 2023)
*Echinochloa crusgalli* var. austro-japonensis	Barnyard grass/Poaceae	Annual herb, strong adaptability, drought resistance, salinity resistance	(Wang et al. 2022)
*Avena sativa* L.	Oats/Poaceae	Annual herb, likes high cold and dry climate, with drought resistance, salinity resistance, barren resistance characteristics	(Zhang et al. 2022)
*Salicornia europaea* L.	Glasswort/Chenopodiaceae	annual succulent herb halophyte	(Ge et al. 2023)
*Medicago sativa* L.	Alfalfa/Fabaceae	Perennial herb, with drought resistance, cold resistance, salinity resistance, strong adaptability, and the effect of improving soil	(Yu et al. 2021)
*Glycyrrhiza uralensis* Fisch.	Liquorice/Fabaceae	Perennial herb, strong adaptability and stress resistance, drought, cold, salt, and alkali resistance	(Gu et al. 2024)

#### Experiment test device, selection, and soil conditions

The experimental test device used for the leaching experiment is as reported earlier (Cheng et al. 2023). Briefly, the main body of the test device includes a plastic basin, a stool with a hollow surface, and a glass bottle for the solution. The outer diameter of the plastic basin was 44 cm, the inner diameter was 38 cm, and the bottom of the basin had three holes. The bottom of the plastic basin had a built-in 300-mesh white nylon filter. The funnel (the upper diameter was 18.5 cm, the lower diameter was 2.5 cm) was also fixed with a 300-mesh white nylon filter. The funnel was inserted through the hollowed stool into a 5 L glass jar with a rubber stopper, and the whole device had a good sealing performance. By setting two types of soil, alkaline soil and saline soil, the soil improvement effect of *E. frumentacea* on Hunan Jizi soil by desalination was studied. The soil with basicity <15% and pH <8.5 is saline, whereas, the soil with basicity >15% and pH > 8.5 is alkaline. Soils from two areas of Qianjin Farm, Xidatan, Pingluo County, Ningxia, and Dongfeng Village, Gaozhuang Township, Pingluo County, Shizuishan City, Ningxia, were selected as the test soils. The basic chemical properties are shown in Table 3.

**Table 3.** Basic chemical properties of saline and alkaline soil used in leaching experiment.

	pH	Basicity (%)	Total salt (g kg^-1^)	Organic matter (g kg^-1^)	Total *N* (%)	Available P (mg kg^-1^)	Available K (mg kg^-1^)	Available N (mg kg^-1^)
Saline soil	8.33	14.6	6.6	4.76	0.023	12.10	159	52
Alkaline soil	9.10	21.75	3.2	6.20	0.014	3.29	240	69

#### Seed sowing

Each plastic pot was filled with 25 kg of soil. The soil was thoroughly mixed before filling. After filling a pot with soil, it was placed on the upper part of the stool with a funnel and watered to soak the soil and left the soil surface to dry slightly. On the 19th of May, hole sowing was carried out. Barnyard grass, barnyard millet, oats, liquorice, and glasswort were planted at a depth of about 2 cm, and alfalfa was planted at a depth of about 4 cm. Barnyard grass, barnyard millet, and oats were sown in 50 holes per pot, 3 seeds per hole, and 45 seedlings per pot (5 cm); glasswort was sown in 30 holes per pot, 3 seeds per hole, and 25 plants per pot (5 cm). Alfalfa was sown in 25 holes per pot, 3 seeds per hole, and 20 plants/pot (5 cm). The management measures are the same throughout the growth period of each treatment. Before June 10, the water is irrigated once a week, and the amount of irrigation is 2 L each time. After June 10, the water was irrigated every 2 days, and the amount of water was 2 L each time.

### Methods/analysis

#### Analysis of root characteristics

Root samples at the seedling stage (25 days after sowing) were collected, washed with water, and put in a plexiglass tray. We used clean water to spread the roots without overlapping and placed them on the EPSON scanner. We used EPSON SCAN scanning software to scan the black and white images such that root scans were black and the background was kept white. Tiff image format was acquired and then we used WinRHIZO Pro 2016a (https://regentinstruments.com) to analyse the root system, and obtain the total root length, average root diameter, total root surface area, root volume, and other indicators of the root system. Root activity was measured using triphenyl tetrazolium chloride. The total root absorption area and active absorption area were measured using the ratio of methylene blue. For dry weight, root samples were over dried at 80 °C for at least 42 hours, and the dry weight was recorded using weighing balance (Singh et al. 2015, Batoolx et al. 2019).

#### Quantification of Ca^2+^, K^+^, Na^+^, Ca^2+^/Na^+^, and K^+^/Na^+^ in roots

The concentrations of Ca^2+^, K^+^, Na^+^, Ca^2+^/Na^+^, and K^+^/Na^+^ in roots were determined by Inductively Coupled Plasma Atomic Emission Spectrometer (ICP-AES, Optima 2100DV, Pekin-Elmer, USA) after digesting in three replicates following the protocol published earlier (Chunthaburee et al. 2016). To prepare the samples, an acid digestion procedure was performed by taking 0.5 g of dried harvested root samples in a small beaker, and 5 ml of HNO_3_ was added to react with plant tissue overnight at room temperature. Next day, the samples were heated carefully until the production of red fumes of NO_2_ stopped. After cooling the suspension, 3 ml of 70% HClO_4_ was added, and samples were heated again and allowed to evaporate until a small volume is left. The samples were diluted with 50 ml distilled water and transferred to 50 ml flasks. Then, the elements were determined by inductively coupled plasma atomic emission spectrometer.

#### Measurement of oxidants and antioxidant enzyme activities in the roots

Roots were analysed for enzymatic antioxidants like POD, SOD, CAT activities, and MDA, proline, and soluble sugars contents following the previously reported protocols (2019).

Estimation of POD activity was done by following the protocol of (1985). One gram of fresh root sample was ground in 10 ml phosphate buffer and centrifuged for 10 min. The clear supernatant was collected. The spectrophotometer was adjusted to read zero at 430 nm and absorbance was taken for 3 min by adding 0.5 ml of 1% H_2_O_2_ in the test cuvette along with plant extract.

The SOD activity was measured by using a previously reported method (1971). About 0.2 g of material was homogenized in 4 ml phosphate buffer (pH 7.8), containing 1 g of polyvinyl pyrrolidone and 0.0278 g of Na_2_EDTA, and then centrifuged at 4 °C for 10 min. The supernatant was collected, and its volume was raised to 8 ml with phosphate buffer of pH 7.1 ml of reaction mixture containing 0.0278 g of Na_2_EDTA, 1.5 g methionine, and 0.04 g of nitro blue tetrazolium chloride in 100 ml phosphate buffer (pH 7.8) and 0.5 ml of reaction mixture containing 0.00113 g riboflavin in 100 ml phosphate buffer (pH 7.8) was mixed with 0.5 ml of enzyme extract. One sample was kept in the light to initiate the reaction at 30 °C for 1 h, while an identical sample was kept in the dark. Absorbance was recorded at 560 nm.

The CAT activity was determined by a previously reported method (1974). About 0.5 g of material was homogenized in 8 ml of phosphate buffer (pH 7) and centrifuged for 10 minutes to collect the supernatant. Then, 3 ml of H_2_O_2_ (2 mM) was mixed with 40 μl of supernatant. By using a spectrophotometer reduction in absorbance by 0.05 units at 240 nm was recorded.

The MDA content was determined by a method reported earlier (2017). About 0.1 g root sample was mixed in 2 ml trichloroacetic acid (0.1%). The homogenate obtained was centrifuged for 15 min at 15 000 g. To the 1 ml supernatant aliquot of the supernatant, 4 ml thiobarbituric acid (0.5%) in 20% TCA was added. The mixture was heated for 30 min at 95 °C, cooled in an ice bath, and centrifuged for 10 min at 10 000 rpm. The absorbance of the supernatant was recorded at 440, 532, and 600 nm.

Proline content was determined by following the method reported earlier (Li et al. 2010, 2018). Briefly, 0.5 g leaf sample was homogenized in 4 ml of 3% sulfosalicylic acid, centrifuged for 10 minutes, and supernatant was collected. Supernatant (2 ml) was mixed with 2 ml of glacial acetic acid and 3 ml of acetic ninhydrin. About 1.25 g of ninhydrin was heated while stirring until dissolved in a solution of 30 ml of glacial acetic acid and 20 ml of 6 M phosphoric acid to create the acetic ninhydrin reagent. After that, the samples were heated to 100 °C for 60 min in a hot water bath. After letting the samples cool down to room temperature, 5 ml of toluene was added to the mixture to extract it. A spectrophotometer was used to measure the absorbance at 520 nm, and toluene was used as a blank. Proline concentration was determined using the standard concentration curve (Supplementary Figure S1) and the formulae reported earlier (Suleiman et al. 2023).

Soluble sugar content was determined by using sugars assay kit/YX-C-B629, and the readings were taken at 540 nm wavelength (Sino best biological technology co, Ltd, Beijing, China) according to the manufacturer's instructions (2021).

### Data analysis

Data organization was performed using Microsoft Excel 2010. Statistical analyses were conducted with DPS 7.05 software, including two-sample Student’s *t*-tests and canonical correlation analysis. Differential analyses were carried out using the least significant difference (LSD) method in two-factor statistical tests. Additionally, one-way ANOVA followed by Tukey’s post-hoc test (*P* < 0.05) was performed using DPS 7.05. For root parameters, one-sample *t*-test was performed using DPS 7.05.

## Results

### Evaluation of *Echinochloa frumentacea* root characteristics under saline–alkali stress

#### Root analysis

**Figure 1.**
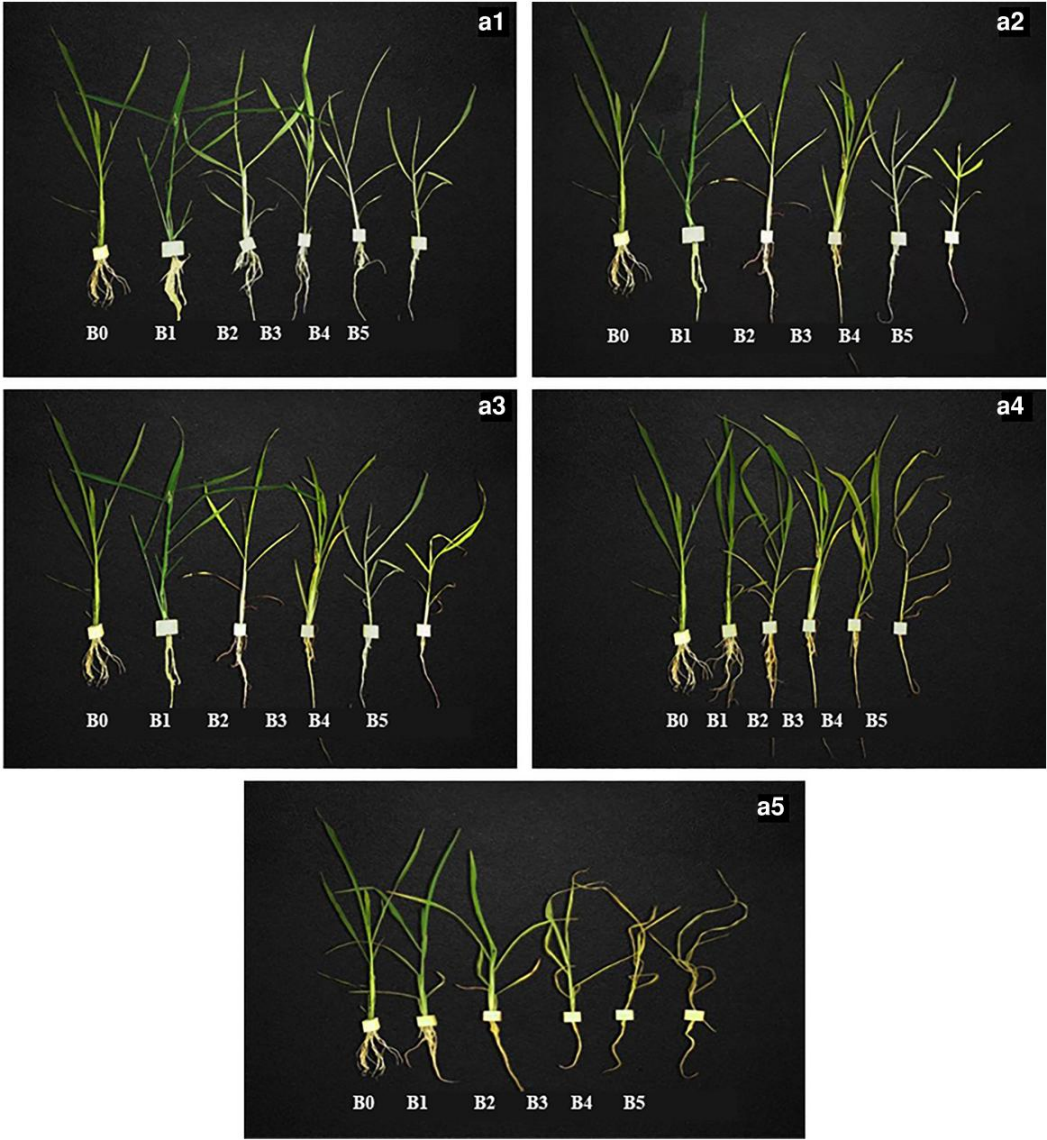
Roots of *Echinochloa frumentacea* (25 days after sowing) under salt stress at 0 mmol L^−1^ (B0), 60 mmol L^−1^ (B1), 120 mmol L^−1^ (B2), 180 mmol L^−1^ (B3), 240 mmol L^−1^ (B4), 300 mmol L^−1^ (B5). Plants were treated with NaCl:Na_2_SO_4_:NaHCO_3_:Na_2_CO_3_ in ratio of 0.5:0.5:0:0 for A1, 0.25:0.5:0.25:0 for A2, 0.05:0.45:0.45:0.05 for A3, 0.1:0.4:0.4:0.1 for A4, and 0.25:0.25:0.25:0.25 for A5.

After salt treatment, roots were analysed for different root parameters (Fig. 1). The maximum root length was observed in plants with 120 mmol L^−1^ of salt treatment of all salt types. Whereas, with the increase of salt concentration, the root length decreased up to 60%%––70% and reached the lowest under 300 mmol L^−1^ salt treatment. Similar results were recorded in the case of root diameter and volume, which showed an increase of about 4.7% at 120 mmol L^−1^ salt treatment. The lowest root diameter and root volume were recorded at 240 and 300 mmol L^−1^ salt treatment in comparison to the control treatment (Table 4).

**Table 4.** Effect on root parameters of *Echinochloa frumentacea* seedlings (25 days after sowing) under salt stress at 0 mmol L^−1^ (B0), 60 mmol L^−1^ (B1), 120 mmol L^−1^ (B2), 180 mmol L^−1^ (B3), 240 mmol L^−1^ (B4), 300 mmol L^−1^ (B5).

Salt groups	Root parameters	A1	A2	A3	A4	A5
B0	Root length (cm)	413.55 ± 16.76b	413.55 ± 16.76b	413.55 ± 16.76b	413.55 ± 16.76b	413.55 ± 16.76c
B1		533.71 ± 14.58a	519.44 ± 12.29a	504.66 ± 15.42a	474.19 ± 22.27ab	448.72 ± 18.72b
B2		542.40 ± 29.06a	530.38 ± 17.14a	528.05 ± 18.92a	511.88 ± 27.31a	490.39 ± 22.55a
B3		361.45 ± 20.51c	353.26 ± 21.74c	347.39 ± 25b	295.37 ± 27.12c	281.44 ± 28.47d
B4		149.82 ± 23.99d	142.95 ± 11.80d	190.84 ± 20.72c	172.80 ± 31.73d	179.95 ± 15.16e
B5		125.33 ± 22.21d	108.55 ± 18.29d	106.00 ± 25.10d	101.31 ± 19.83d	93.77 ± 13.83f
B0	Root diameter (mm)	0.490 ± 0.02a	0.490 ± 0.02a	0.490 ± 0.02a	0.490 ± 0.02a	0.490 ± 0.02a
B1		0.492 ± 0.02a	0.490 ± 0.02a	0.486 ± 0.01a	0.480 ± 0.01ab	0.471 ± 0.01a
B2		0.511 ± 0.02a	0.487 ± 0.02a	0.482 ± 0.01a	0.478 ± 0.01a	0.466 ± 0.01a
B3		0.475 ± 0.02a	0.458 ± 0.01a	0.449 ± 0.00a	0.435 ± 0.01b	0.417 ± 0.01b
B4		0.353 ± 0.03b	0.334 ± 0.02b	0.311 ± 0.03b	0.302 ± 0.02c	0.238 ± 0.01c
B5		0.327 ± 0.04b	0.318 ± 0.02b	0.272 ± 0.04b	0.239 ± 0.01d	0.229 ± 0.01c
B0	Root volume (cm^3^)	0.783 ± 0.07b	0.783 ± 0.07b	0.783 ± 0.07b	0.783 ± 0.07b	0.783 ± 0.07ab
B1		0.953 ± 0.01a	0.937 ± 0.03a	0.915 ± 0.02a	0.884 ± 0.02ab	0.842 ± 0.02a
B2		1.008 ± 0.12a	0.966 ± 0.01a	0.947 ± 0.02a	0.919 ± 0.01a	0.835 ± 0.02a
B3		0.794 ± 0.04b	0.728 ± 0.03b	0.664 ± 0.02b	0.647 ± 0.07c	0.629 ± 0.06b
B4		0.544 ± 0.02c	0.525 ± 0.02c	0.489 ± 0.04c	0.468 ± 0.03d	0.331 ± 0.06c
B5		0.517 ± 0.01c	0.499 ± 0.02c	0.473 ± 0.02c	0.253 ± 0.03e	0.277 ± 0.01c
B0	Total absorbing area (cm^2^)	63.81 ± 3.78ab	63.81 ± 3.78a	63.81 ± 3.78a	63.81 ± 3.78a	63.81 ± 3.78a
B1		65.18 ± 5.26ab	63.63 ± 8.12a	61.50 ± 5.11ab	57.40 ± 2.54ab	53.75 ± 2.94b
B2		76.75 ± 5.19a	68.44 ± 7.55a	55.87 ± 4.17ab	54.06 ± 2.06bc	50.68 ± 3.76bc
B3		57.75 ± 6.11b	53.93 ± 7.68a	49.90 ± 4.36b	46.28 ± 3.68c	42.84 ± 3.14c
B4		33.49 ± 5.17c	30.72 ± 4.53b	26.74 ± 4.00c	22.85 ± 2.61d	19.92 ± 2.22d
B5		28.52 ± 4.79c	24.79 ± 4.19b	21.72 ± 2.24c	16.37 ± 2.4d	14.51 ± 1.94d
B0	Active absorption area (cm^2^)	45.39 ± 4.00ab	45.39 ± 4.00b	45.39 ± 4.00a	45.39 ± 4.00a	45.39 ± 4.00a
B1		52.9 ± 4.26a	46.31 ± 3.84ab	43.59 ± 3.49a	21.02 ± 1.89b	19.61 ± 3.53b
B2		55.37 ± 2.4a	52.40 ± 2.56a	40.71 ± 2.80a	20.49 ± 2.55b	16.39 ± 4.38bc
B3		38.55 ± 3.67b	31.94 ± 4.24c	24.41 ± 2.98b	18.54 ± 3.57b	10.75 ± 3.31c
B4		19.63 ± 3.11c	18.26 ± 2.79d	17.64 ± 2.55bc	6.66 ± 1.33c	0.00 ± 0.00d
B5		17.48 ± 3.03c	15.17 ± 2.40d	14.05 ± 1.98c	0.00 ± 0.00d	0.00 ± 0.00d
B0	Root activity µg (mg h)^−1^	0.042 ± 0.002b	0.042 ± 0.002a	0.042 ± 0.002a	0.042 ± 0.002a	0.042 ± 0.002a
B1		0.044 ± 0.001ab	0.042 ± 0.002a	0.040 ± 0.001a	0.039 ± 0.001a	0.037 ± 0.002ab
B2		0.048 ± 0.003a	0.047 ± 0.002a	0.045 ± 0.002a	0.043 ± 0.001a	0.035 ± 0.001b
B3		0.027 ± 0.002c	0.025 ± 0.002b	0.025 ± 0.001b	0.021 ± 0.001b	0.017 ± 0.002c
B4		0.016 ± 0.001d	0.014 ± 0.001c	0.012 ± 0.002c	0.01 ± 0.001c	0.00 ± 0.00d
B5		0.014 ± 0.001d	0.011 ± 0.001c	0.010 ± 0.001c	0.00 ± 0.00d	0.00 ± 0.00d
B0	Root fresh weight (g)	0.227 ± 0.02bc	0.227 ± 0.02bc	0.227 ± 0.02b	0.227 ± 0.02b	0.227 ± 0.02a
B1		0.235 ± 0.01b	0.249 ± 0.01ab	0.24 ± 0.01b	0.236 ± 0.01b	0.218 ± 0.02ab
B2		0.290 ± 0.01a	0.285 ± 0.02a	0.277 ± 0.01a	0.268 ± 0.02a	0.205 ± 0.00ab
B3		0.201 ± 0.03cd	0.198 ± 0.01cd	0.195 ± 0.01c	0.188 ± 0.03c	0.184 ± 0.01bc
B4		0.170 ± 0.01de	0.167 ± 0.01d	0.165 ± 0.01d	0.161 ± 0.02d	0.155 ± 0.02cd
B5		0.167 ± 0.01e	0.164 ± 0.01d	0.161 ± 0.01d	0.142 ± 0.01d	0.139 ± 0.02d
B0	Root dry weight (g)	0.024 ± 0.004b	0.024 ± 0.004bc	0.024 ± 0.004ab	0.024 ± 0.004a	0.024 ± 0.004a
B1		0.029 ± 0.002ab	0.027 ± 0.002ab	0.024 ± 0.002ab	0.022 ± 0.002a	0.023 ± 0.002a
B2		0.032 ± 0.002a	0.03 ± 0.002a	0.027 ± 0.004a	0.017 ± 0.001b	0.020 ± 0.002b
B3		0.026 ± 0.003ab	0.023 ± 0.001bc	0.02 ± 0.002bc	0.015 ± 0.002b	0.019 ± 0.002b
B4		0.024 ± 0.002b	0.02 ± 0.002c	0.016 ± 0.001cd	0.014 ± 0.001b	0.013 ± 0.002c
B5		0.017 ± 0.001c	0.015 ± 0.001d	0.011 ± 0.002d	0.009 ± 0.001c	0.006 ± 0.001d

For each treatment, values are given as mean ± standard deviation. Different lowercase letters indicate significant differences among the treatments, as measured by one-way ANOVA followed by Tukey’s test (*P* < 0.05).

The total surface area of the roots also showed the same trend of increase in the case of A1 and A2 salts at 120 mmol L^−1^ whereas in the case of A3, A4, and A5, the surface area gradually decreased with the increase of salt concentration. In A1, A2, and A3, the plants tolerated and maintained the absorption area up to 120 mmol L^−1^ concentration (Table 4).

The active absorption area after the salt application was also seen to be gradually decreasing with the increase of salt concentration, indicating that plants have the potential to tolerate up to 120 mmol L^−1^ of salt stress. In the case of root activity, plants tolerated the stress to maintain it at 120 mmol L^−1^ but as the salt concentration increased, the root activity started to decrease (Table 4).

In the end, root fresh weight and dry weight were estimated which showed a similar trend with no significant differences at 60, 120, and 180 mmol L^−1^ of salt concentrations when compared with control. However, the weight started to decrease as the salt concentration was increased up to 240 and 300 mmol L^−1^ (Table 4).

These results show that *E. frumentacea* can perform significantly better when the salt concentrations are 60–120 mmol L^−1^, suggest that the species has an adaptive strategy to increasing salt concentration (Zhang et al. 2024).

For each treatment, values are given as mean ± standard deviation. Different lowercase letters indicate significant differences among the treatments, as measured by one-way ANOVA followed by Tukey’s test (*P* < 0.05).

pH values were also recorded, and it revealed that there was a gradual increase with the increase in salt concentrations (Table 5).

**Table 5.** pH values of roots recorded after salt treatment at 60 mmol L^−1^ (B1), 120 mmol L^−1^ (B2), 180 mmol L^−1^ (B3), 240 mmol L^−1^ (B4), and 300 mmol L^−1^ (B5).

Salt treatment		pH	Linear regression equation	*R* ^2^	pH suitable	pH critical	pH limit
60	A1	7.02	*y* = −13.810*x* + 155.013	0.827*	8.34	9.3	10.83
	A2	8.27					
	A3	8.63					
	A4	9.25					
	A5	9.62					
120	A1	7.03	*y* = −15.769*x* + 172.808	0.797*	8.45	9.2	10.61
	A2	8.4					
	A3	8.66					
	A4	9.27					
	A5	9.68					
180	A1	7.09	*y* = −10.316*x* + 113.737	0.904*	8.26	9.17	10.62
	A2	8.46					
	A3	8.74					
	A4	9.3					
	A5	9.7					
240	A1	7.1	*y* = −6.689*x* + 71.02	0.895*	8.23	8.92	10.01
	A2	8.45					
	A3	8.71					
	A4	9.35					
	A5	9.73					
300	A1	7.12	*y* = −7.365*x* + au73.355	0.774	8.18	8.77	9.72
	A2	8.44					
	A3	8.7					
	A4	9.44					
	A5	9.76					

The molar ratios of NaCl:Na_2_SO_4_:NaHCO_3_:Na_2_CO_3_ were 0.5:0.5:0:0 for A1, 0.25:0.5:0.25:0 for A2, 0.05:0.45:0.45:0.05 for A3, 0.1:0.4:0.4:0.1 for A4, and 0.25:0.25:0.25:0.25 for A5. *means significant correlation at *P* < 0.05.

#### Quantification of Ca^2+^, K^+^, Na^+^, Ca^2+^/Na^+^, and K^+^/Na^+^ in roots

**Figure 2.**
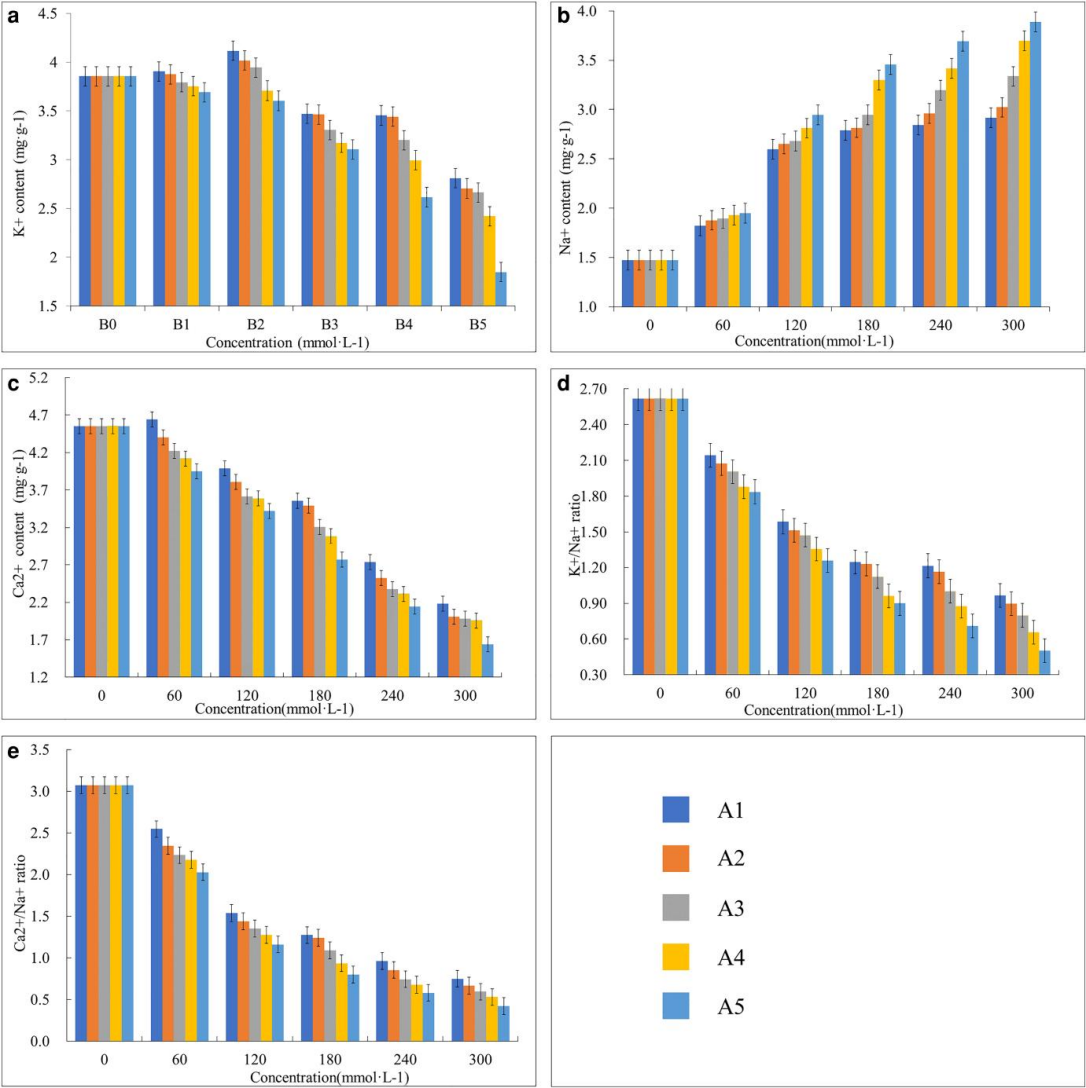
Analysis for absorbed ions; (a) K^+^, (b) Na^+^, (c) Ca^2+^, (d) K^+^:Na^+^, and (e) Ca^2+^:Na^+^ by roots under salt stress at 0 mmol L^−1^ (B0), 60 mmol L^−1^ (B1), 120 mmol L^−1^ (B2), 180 mmol L^−1^ (B3), 240 mmol L^−1^ (B4), 300 mmol L^−1^ (B5). For each treatment, the bars represent mean values, and the error bars represent ± standard error.

The concentration of ions absorbed by the roots was estimated after salts treatment. The absorption of K^+^ increased at 60 and 120 mmol L^−1^ and then gradually decreased with the increase in salt concentration. In contrast, the absorption capacity of Na^+^ by roots showed an increasing trend with the increase of salt concentration as compared to control. The trend is opposite in the case of Ca^2+^ absorption, where a decrease was observed with the increase in salt concentration (Fig. 2).

The potassium–sodium (K^+^/Na^+^) ratio was higher in non-stressed plants and starts to gradually decrease after salt treatment. Similar results were recorded in the Ca^2+^/Na^+^ (Fig. 2).

#### Antioxidant enzyme activities, MDA, proline, and soluble sugar content

##### Peroxidase activity

**Figure 3.**
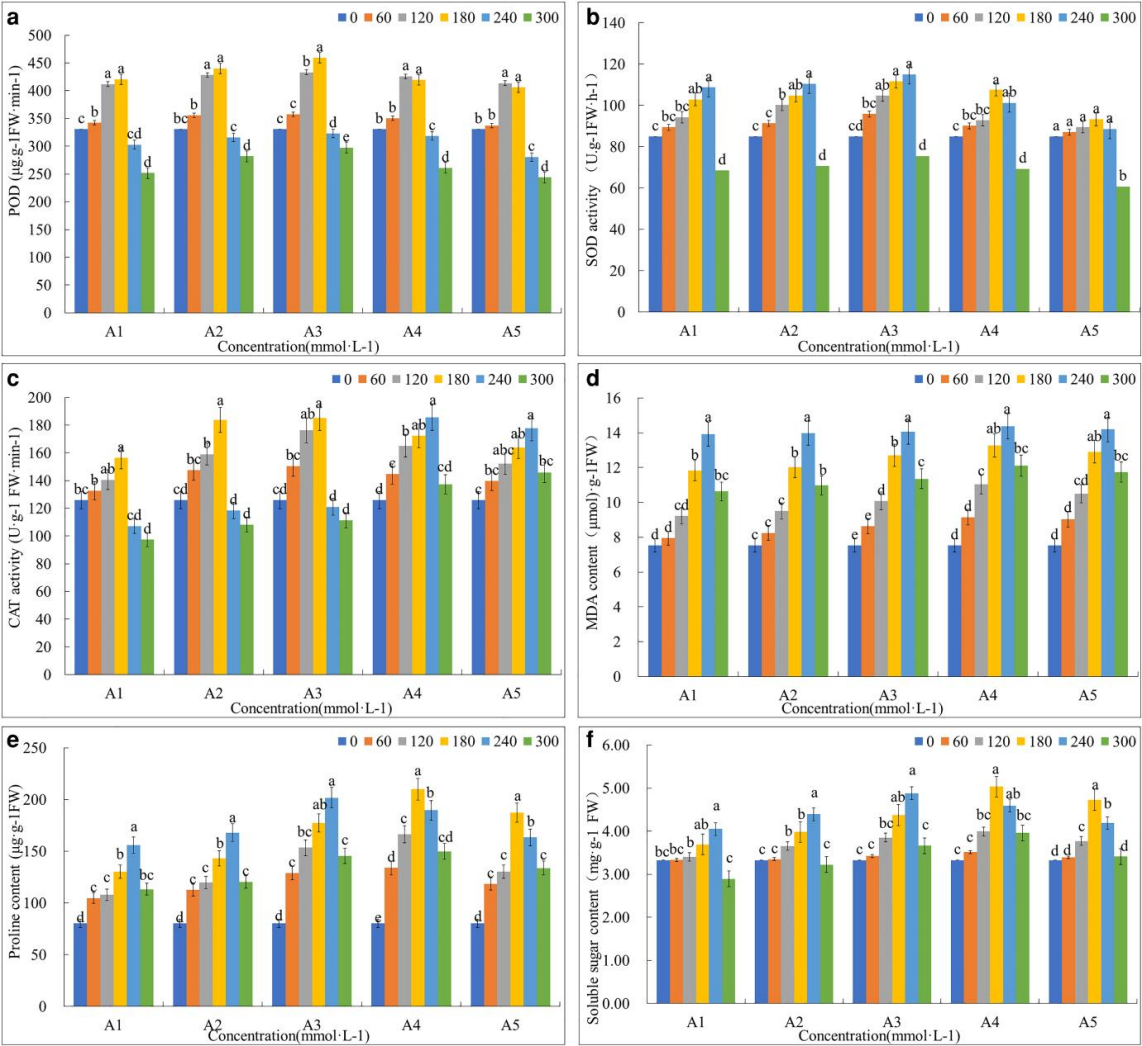
Antioxidant enzyme levels (a) POD, (b) SOD, (c) CAT, (d) MDA, (e) proline, and (f) soluble sugar contents of *Echinochloa frumentacea* root under salt stress at 60 mmol L^−1^ (B1), 120 mmol L^−1^ (B2), 180 mmol L^−1^ (B3), 240 mmol L^−1^ (B4), and 300 mmol L^−1^ (B5), compared to control (B0). Different lowercase letters above each bar indicate significant differences among the treatments, as measured by one-way ANOVA followed by Tukey’s test (*P* < 0.05). The bars represent mean values ± standard error.

The POD activity in roots increased under salt stress conditions at 60, 120, and 180 mmol L^−1^ compared to control, and the highest POD activity was recorded as 31.25%, 33%, and 38.9% at 180 mmol L^−1^ in the case of A1, A2 and A3, respectively. In contrast, in the case of A4 and A5, the highest POD activity of 28.8% and 25% was recorded at 120 mmol L^−1^ concentration. The POD activity decreased at 180 and 300 mmol L^−1^ salt concentrations up to 8 (−−10%) and 20 (−−25%) and the lowest was recorded as −−26% in A5 at 300 mmol L^−1^ of salt concentration in comparison to control (Fig. 3).

##### Superoxide dismutase activity

The SOD activity in roots showed an increasing trend with the increase in salt concentration from 60 mmol L^−1^ reaching the highest point of 34.97%, at 240 mmol L^−1^ in A3 as compared to control. There were no significant differences in SOD activity in the case of A5 at all salt concentrations. At the highest salt concentration of 300 mmol L^−1^, a significant decrease of about 21.2% in SOD activity was seen in all treatments (Fig. 3).

##### Catalase activity

Similarly, an increase in catalase activity by roots was seen with the increase of salt concentration to 180 mmol L^−1^ in A1, A2, and A3 and to 240 mmol L^−1^ in A4 and A5. The highest catalase activity was recorded as 41.1% in A3 at 180 mmol L^−1^ and the lowest was recorded, 22% in A1 at 300 mmol L^−1^ in comparison with control (Fig. 3).

##### Malondialdehyde content

The MDA content in roots showed an increasing trend with the increase in salt concentrations. The highest was recorded as 58%, 62%, 66%, 70.7%, and 90.7% at 300 mmol L^−1^ in A1, A2, A3, A4, and A5, respectively. All treatments showed a significant increase in MDA at all concentrations of salt as compared to control (Fig. 3).

##### Proline content

Proline content also shows a similar trend of increasing from 60 to 240 mmol L^−1^ in A1, A2, and A3 and from 60 to 180 mmol L^−1^ in A4 and A5. About 161-fold of increase was recorded in A4 at 120 mmol L^−1^ salt concentration. At 300 mmol L^−1^ salt treatment, a sudden significant decrease was recorded among all treatments as compared to other salt concentrations, but it was still higher as compared to control (Fig. 3).

##### Soluble sugar content

Soluble sugar content showed a significant increase at 180 and 240 mmol L^−1^ in all treatments with the highest 21%, 31.83%, and 45% in A1, A2, and A3, respectively, at 240 mmol L^−1^ and 51% and 42% at 180 mmol L^−1^ concentration in A4 and A5, respectively. A significant decrease in sugar content was recorded at 300 mmol L^−1^ concentration in A1 and A2 as compared with control (Fig. 3).

### Effects of salt absorption and leaching by the roots of the six forage species

The fibrous root system of *E. frumentacea* can penetrate about 40 cm into the soil. Its interpenetration and expansion effects are strong, and the surface area and active absorption area are increased, thereby promoting the downward leaching of salt and the absorption of salt by the root system (Table 6). Canonical correlation analysis showed that with the increase of the total root length and root volume of the root system, more Na^+^, Cl^−^, and SO_4_^2−^ were leached. The higher the vitality, the more the amount of Na^+^ and SO_4_^2−^ absorbed. The average student *t*-test of the two groups showed that the absorption of the eight major ions was significantly higher in the saline soil than in the alkaline soil. Compared with the alkaline soil, the total amount of ions and absorbed ions was 246.75% and 26.19% higher in saline soil, respectively. The amounts of Na^+^, Cl^−^, SO4^2−^, CO3^2−^, and HCO_3_^−^ leaching in the saline soil were significantly higher than those in the alkaline soil by 69.55%, 74.36%, 60.80%, 82.88%, and 85.94%, respectively. Similarly, the absorption of Na^+^, Cl^−^ as well as the amount of SO_4_^2−^ were 18.44%, 21.05%, and 7.37% higher the saline soil. Through a two-factor randomized block-group trial comparison study, it was found that the leaching of Na^+^, K^+^, Ca^2+^, Cl^−^, and SO_4_^2−^ in the six forage grasses had significant differences among saline–alkaline land types, pasture types, and saline–alkaline land types × pasture types. In addition to Mg^2+^, other salt ions absorbed by the roots of the six forage species, K^+^/Na^+^ and Ca^2+^/Na^+^ were significantly different among saline–alkaline land types, pasture types, and saline–alkaline land type × pasture type interactions (Table 7).

**Table 6.** Root parameters of 25 days old *Echinochloa frumentacea* grown under saline and alkaline soil.

Root parameters	Saline alkali soil type	Average value	Standard error	*T* value	Sig double tail detection
Total root length (cm/plant)	Saline soil	201.13	11.270	3.185	0.032
	Alkaline soil	158.64	7.142		
Average diameter (mm/plant)	Saline soil	4.03	0.058	6.045	0.004
	Alkaline soil	2.63	0.219		
Root volume (cm^3^/plant)	Saline soil	34.36	1.861	3.094	0.036
	Alkaline soil	24.97	2.399		
Root fresh weight (g/plant)	Saline soil	0.33	0.020	3.207	0.033
	Alkaline soil	0.25	0.015		
Root dry weight (g/plant)	Saline soil	0.04	0.032	3.569	0.023
	Alkaline soil	0.03	0.023		
Total root absorption area (cm^2^/plant)	Saline soil	116.75	15.001	2.841	0.047
	Alkaline soil	95.66	5.499		
Active absorption area (cm^2^/plant)	Saline soil	60.30	3.382	2.784	0.050
	Alkaline soil	47.55	2.952		
Root activity (μg mg h)^−1^	Saline soil	0.04	0.007	3.608	0.023
	Alkaline soil	0.04	0.003		

**Table 7.** Quantification of absorbed ions in g kg^−1^ by the roots of the six forage species grown under saline and alkaline conditions.

Soil type	Species	Na^+^	K^+^	Ca^2+^	Mg^2+^	Cl^−^	SO_4_^2−^	K^+^/Na^+^	Ca^2+^/Na^+^
Saline	*E. frumentacea*	4.99 ± 0.13b	3.94 ± 0.21b	3.2 ± 0.06a	0.100 ± 0.015a	0.019 ± 0.002b	6.19 ± 0.17a	0.79 ± 0.025a	0.64 ± 0.04b
	*E. crusgalli*	3.51 ± 0.03fg	2.56 ± 0.12e	1.95 ± 0.05d	0.078 ± 0.006f	0.008 ± 0.0005de	4.75 ± 0.12d	0.73 ± 0.02bc	0.56 ± 0.05c
	*A. sativa*	3.23 ± 0.03g	2.31 ± 0.03f	2.45 ± 0.04b	0.099 ± 0.006a	0.006 ± 0.0008def	5.63 ± 0.07b	0.72 ± 0.02bc	0.76 ± 0.05a
	*S. europaea*	8.29 ± 0.44a	5.83 ± 0.14a	2.48 ± 0.04b	0.054 ± 0.003h	0.069 ± 0.008a	3.25 ± 0.20g	0.70 ± 0.02cd	0.3 ± 0.02e
	*M. sativa*	4.40 ± 0.02cd	3.05 ± 0.07c	2.53 ± 0.03b	0.080 ± 0.007ef	0.006 ± 0.0004def	5.52 ± 0.03b	0.69 ± 0.03cd	0.58 ± 0.03c
	*G. uralensis*	4.53 ± 0.13c	2.96 ± 0.06c	2.27 ± 0.09c	0.067 ± 0.003g	0.004 ± 0.0006f	5.10 ± 0.18c	0.65 ± 0.02ef	0.50 ± 0.03d
Alkali	*E. frumentacea*	4.07 ± 0.04de	3.05 ± 0.06c	0.9 ± 0.04fg	0.090 ± 0.007cd	0.015 ± 0.002c	5.20 ± 0.03c	0.75 ± 0.02b	0.22 ± 0.02gh
	*E. crusgalli*	4.03 ± 0.06de	2.53 ± 0.07e	0.56 ± 0.02h	0.057 ± 0.006h	0.009 ± 0.001d	4.40 ± 0.2f	0.63 ± 0.006f	0.14 ± 0.03i
	*A. sativa*	3.80 ± 0.08ef	2.75 ± 0.05d	0.95 ± 0.04f	0.097 ± 0.004ab	0.004 ± 0.001f	4.63 ± 0.14e	0.72 ± 0.025bcd	0.25 ± 0.02fg
	*S. europaea*	2.57 ± 0.06h	3.94 ± 0.04b	0.56 ± 0.02h	0.045 ± 0.003i	0.005 ± 0.001ef	4.57 ± 0.05e	0.68 ± 0.02de	0.22 ± 0.02gh
	*M. sativa*	3.82 ± 0.07ef	2.56 ± 0.08e	1.06 ± 0.08e	0.092 ± 0.002bc	0.004 ± 0.0004f	4.62 ± 0.04e	0.55 ± 0.03g	0.28 ± 0.02ef
	*G. uralensis*	4.16 ± 0.04cde	2.31 ± 0.04f	0.86 ± 0.03g	0.085 ± 0.005de	0.004 ± 0.0022f	4.35 ± f	0.49 ± 0.07h	0.21 ± 0.03h

For each, values are means. Different lower case letters indicate significant differences among species (*P* < 0.05).

It can be seen that *E. frumentacea* is very suitable for growth in saline soils, especially in sulfate soils, based on its high grass yield, strong salt tolerance, and strong Na^+^ and SO_4_^2−^ accumulation characteristics. Moreover, the root system of *E. frumentacea* can be used to dechlorinate the chloride saline–alkaline soil and reduce the toxic effect of Cl^−^ on plants (Table 7).

## Discussion

Globally, agriculture faces many challenges: producing 70% more food for an additional 2.3 billion people by 2050, while fighting poverty and hunger, using scarce natural resources more efficiently, and adapting to climate change (Food and Agriculture Organization of the United Nations (FAO) 2009). However, crop productivity is not increasing in parallel with the food demand. In most cases, lower productivity is due to various abiotic stresses. Reducing crop losses due to various environmental stresses is a major concern to meet the increasing food demand (Shanker and Venkateswarlu 2011). Mechanisms of salt tolerance in species such as *E. frumentacea* are not yet fully understood. Although, earlier work have highlighted that *E. frumentacea* is a salt tolerant species and can be grown on salt affected soils (Cheng et al. 2023). Such mechanisms in plant species can be explained to some extent by evaluating the stress adaptation effectors that mediate ion homeostasis, osmolyte biosynthesis, radical scavenging ability, water transport, and long-distance response coordination (Hasegawa et al. 2000). However, limited or no attempts have been made to improve the yield of *E. frumentacea* under stress conditions. In this regard, our study investigates the responses and tolerance level of *E. frumentacea* to saline–alkaline environment by providing useful data related to possible mechanisms by which growth, development and physiology of plants are affected by salinity.

The effects of salt absorption and leaching by roots of *E. frumentacea* under salinity stress revealed differences in salinity tolerance depending upon salt concentration and types. These differences were noted by evaluating the physiological and protective enzyme parameters. Under salinity stress, root growth measured in terms of fresh weight and dry weight increased up to 120 mmol L^−1^ but considerably reduced at higher salt concentrations of 180, 240, and 300 mmol L^−1^. Previous work on *E. frumentacea* had also indicated that the species adapts by gradually improving traits such as root thickness, root hairs, and density (Zhang et al. 2024). Similar results were recorded in the case of root volume, density, absorption area, root diameter, and active area, which showed an increase to 120 mmol L^−1^ but significantly decreased as the salts concentration increased up to 180, 240, and 300 mmol L^−1^. Our findings of the root characteristics of *E. frumentacea* in saline–alkaline soil are consistent with the earlier work (2010), which reported that salt tolerance levels decreased as the salts concentration increased in rice at the seedling stage. Similarly, in rice, it was reported that salt stress significantly reduced the total dry matter of the plant at the seedling stage (2010). Also in rice, Ahmad and Prasad (2012) observed that salt stress (of 50 mM NaCl) caused a significant decrease in both fresh and dry weights of the salt-sensitive variety IR29 at the seedling stage.

*Echinochloa frumentacea* is well known for its ability to thrive on salt-affected soils (Singh et al. 2021). Salt tolerance in *E. frumentacea* varies considerably across genotypes, which is due to different levels of ion homeostasis strategies that the species has evolved to cope with excess Na^+^ (Blumwald 2000). In the current study, the K^+^/Na^+^ ratio decreased and Na^+^ increased with higher salt concentration. This indicates the low tolerance level of *E. frumentacea* under extreme salt stress. The findings in our study are in agreement with those conducted on rice (2012), which observed that the tolerant rice cultivar FL478 was able to maintain a significantly higher K^+^/Na^+^ ratio in comparison to the sensitive cultivar IR29, which had a lower K^+^/Na^+^ ratio in roots during their exposure to salt stress. In *E. frumentacea*, a 4-year-old study (Lu 2021) proved that high absorption capacity of K^+^/Na^+^ results in more tolerance to salt stress.

Apart from ion homeostasis, proline accumulation is another well-known mechanism that has been triggered to cope with drought or salinity stress in several plant species. Proline plays crucial role in protecting the subcellular structures and mediating osmotic adjustment in stressed conditions (Parvaiz and Satyawati 2008). Proline serves as an energy source and hydroxyl radical scavenger (Munns and Tester 2008). We observed an increase in proline content with the increase of salt stress concentration up to 161-folds at 180 mmol L^−1^. Increased level of proline via upregulation of proline biosynthesis pathway keeps plants safe from stress by membrane protection and maintaining cell water content (Sandhya et al. 2010). Our findings are in line with a recent study (Lu 2021), which stated that an increase in proline content helps plants to develop tolerance against salt stress. We also estimated the activity of protective antioxidant enzymes like SOD, POD, and CAT and observed a similar increasing trend in their production with the increase in the salinity concentration to 240 mmol L^−1^ followed by a sudden decrease at 300 mmol L^−1^. The enhanced activities of defense-related enzymes contributed to the protection of plants against several stress conditions. Stress resistance is strongly correlated to antioxidant enzyme activity (Bano and Muqarab 2017). Production and scavenging of ROS are balanced by different antioxidant enzymes like SOD, POD, CAT, etc. Our results on the increased levels of SOD, POD, and CAT under stress conditions are consistent with those of the earlier study (2005), which stated that tomato plants under biotic stress resulted in an increment of SOD level in leaves. Similarly, an increased POD activity under stress conditions have been reported (2017). Another study (Sozharajan and Natarajan 2013) reported that an increase in antioxidant enzyme activity in response to cellular damage caused by salt stress enables plants to tolerate salt stress. Our results on the continuous increase in MDA content with the increase in salt concentration are also valuable. The low MDA content is commonly considered the best physiological component of stress tolerance in plants (Singh et al. 2021). Therefore, our results indicate the reduction in tolerance level of *E. frumentacea* under higher salt concentrations. Similar results have been previously reported in *E. frumentacea* (2021). In case of maize, a study (2011) reported that the content of MDA activity was found to be higher in sensitive maize genotype as compared to tolerant one. The unchanged MDA level is a characteristic of the tolerant plant which is better equipped with a better free radical scavenging system as compared with a sensitive group of plant that offered protection against oxidative stress (Singh et al. 2021).

Our results on the comparison of *E. frumentacea* with *E. crusgalli*, *A. sativa*, *S. europaea*, *M. sativa*, *and G. uralensis*, our results showed that the amount of Na^+^, Cl^−^, SO_4_^2−^, CO_3_^2−^, and HCO_3_^−^ leaching in the saline soil was significantly higher than that in the alkaline soil planting. Interestingly, similar results had been previously observed in the case of salt–tolerant wheat cultivars at the seedling stage (Esfandiari et al. 2011). Our observations on the better leaching of ions by *E. frumentacea* could be due to its ability to absorb higher ions than other plant species (Cheng et al. 2023). *Echinochloa frumentacea* showed higher tolerance in saline soil with decreased Na^+^ and increased K^+^/Na^+^ as compared to other species and alkaline soil. Similarly, several previous studies have suggested that the K^+^/Na^+^ ratios can be used as an important physiological selection criterion for salt tolerance in many plant species such as tomato, chickpea, and barley (Chunthaburee et al. 2016) and *E. frumentacea* (Lu et al. 2021). To further elaborate the mechanism of ion absorption in the roots of *E. frumentacea*, comparative transcriptomic studies would be helpful in revealing the possible mechanisms, genes (and networks), and pathways.

## Conclusion

In our study, we establish that the *E. frumentacea* can tolerate salt concentrations of 60–120 mmol L^−1^, while the higher salt concentrations i.e. 240–300 mmol L^−1^ can significantly affect the root morphology. Consistently, the K^+^ absorption increases till 120 mmol L^−1^ and then decreases. Our results indicate that both the K^+^/Na^+^ and Ca^2+^/Na^+^ ratios decrease in stressed plant roots compared to that of control. The higher K^+^/Na^+^ ratio during 60–120 mmol L^−1^ salt concentrations indicate the ability of *E. frumentacea* to tolerate these levels and gradual decrease suggests reducing stress tolerance. The increasing trend of the relative antioxidant enzyme activities concludes that *E. frumentacea* uses antioxidant enzymes to scavenge the radicals produced during salt stress. The comparison between alkaline and saline soils indicates that the absorption of the six major ions was significantly higher in the latter type of soil than the former one. Hence, our results propose that *E. frumentacea* is very suitable for growth in saline soils, especially in sulfate-affected soils, based on its yield, strong salt tolerance, and strong Na^+^ and SO_4_^2−^ accumulation characteristics.

## Supplementary Material

plaf066_Supplementary_Data

## Data Availability

All the data related to this work is given within the manuscript or as Supplementary material.
