# Correction to: Evaluation of *Echinochloa frumentacea* under saline–alkaline conditions and its comparison with five forage species

**DOI:** 10.1093/aobpla/plag007

**Published:** 2026-02-20

This is a correction to: Xueqin Wang, Qingxia Zhang, Fengxia Li, Qiaoli Ma, Bo Zhang, Fengju Zhang, Evaluation of *Echinochloa frumentacea* under saline–alkaline conditions and its comparison with five forage species, *AoB PLANTS*, Volume 17, Issue 6, December 2025, plaf066, https://doi.org/10.1093/aobpla/plaf066

In the originally published version of the paper, only one affiliation was published in the PDF version. The correct affiliations have now been assigned to the respective authors and listed below the author list in the PDF version.

