# Supplementary figures and images for: Evaluation of Echinochloa frumentacea under saline–alkaline conditions and its comparison with five forage species
Source: AoB Plants. 2025 Nov 20;17(6):plaf066. doi: 10.1093/aobpla/plaf066 (PMC12673843; doi:10.1093/aobpla/plaf066)

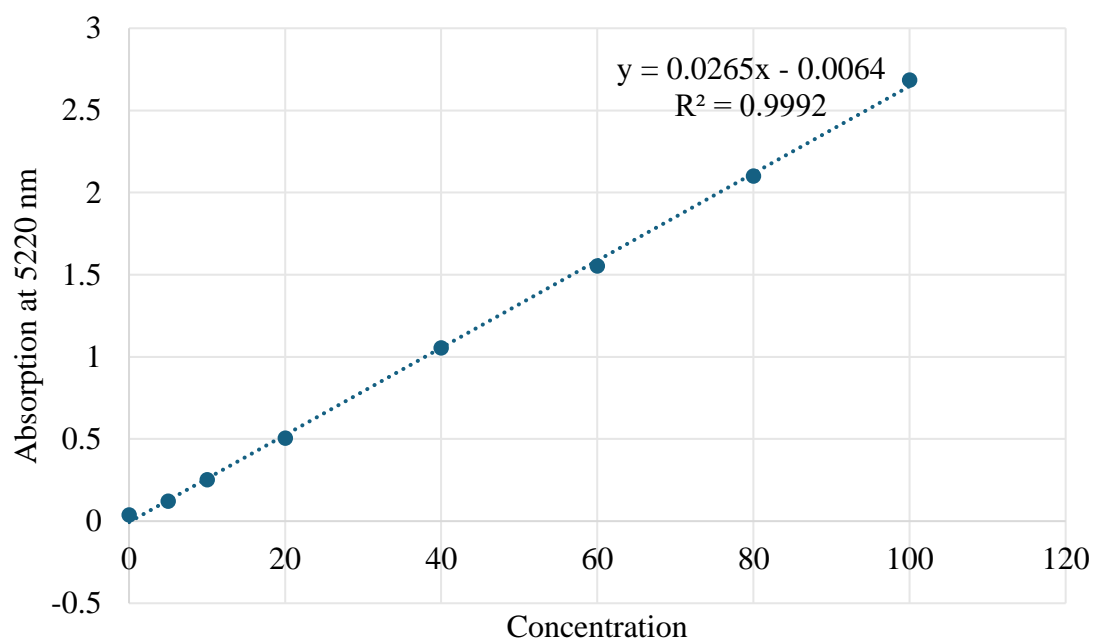

**Supplementary Figure 1.** Calibration curve for evaluation of proline content.

Supplement: plaf066_Supplementary_Data [file plaf066_supplementary_data.pdf]
